# Supplementary material for: Prevalence and morphometry of the fabella in cadaveric and radiological studies: Is there a link to knee osteoarthritis?
Source: PLoS One. 2026 Jan 5;21(1):e0340399. doi: 10.1371/journal.pone.0340399 (PMC12768361; doi:10.1371/journal.pone.0340399)
Supplement: Attachment — Submitted filename: PONE-D-25-50561.pdf [file pone.0340399.s004.pdf]

PLOS ONE

PREVALENCE AND MORPHOMETRY OF THE FABELLA IN CADAVERIC AND RADIOLOGICAL STUDIES: IS THERE A LINK TO KNEE OSTEOARTHRITIS?

--Manuscript Draft--

**Manuscript Number:**	PONE-D-25-50561
**Article Type:**	Research Article
**Full Title:**	PREVALENCE AND MORPHOMETRY OF THE FABELLA IN CADAVERIC AND RADIOLOGICAL STUDIES: IS THERE A LINK TO KNEE OSTEOARTHRITIS?
**Short Title:**	FABELLA PREVALENCE AND OSTEOARTHRITIS
**Corresponding Author:**	Ebru YOLACAN<br>Samsun University: Samsun Universitesi<br>ATAKUM, SAMSUN TÜRKIYE
**Keywords:**	fabella; sesamoid bone; Turkish population; Osteoarthritis
**Abstract:**	Objective – To investigate the morphometric features of the fabella in cadaveric specimens and evaluate its prevalence and potential association with knee osteoarthritis through radiological analysis.<br>Methods – In the cadaveric study, 65 knees from 32 cadavers and 3 leg specimens were dissected. Fabellae were measured and analyzed histologically. In the radiological study, bilateral knee X-rays of 1712 patients with osteoarthritis and 2304 control subjects were retrospectively reviewed. The presence and laterality of the fabella were recorded.<br>Results – Fabella was detected in 18.5% of dissected knees. No significant sex-based differences were found in morphometric measurements (p>0.05). In the radiological study, the overall prevalence was 36.5% (37.6% in patients, 35.7% in controls), with no statistically significant association between fabella presence and osteoarthritis (p>0.05). Lateralization patterns did not differ between groups.<br>Conclusion – This study provides the first morphometric data on fabellae in Turkish cadavers and suggests no significant relationship between fabella presence and knee osteoarthritis. While fabella is a common anatomical variant, its clinical relevance in osteoarthritis remains inconclusive, warranting further investigation.
**Order of Authors:**	Ebru YOLACAN
	Nurcan ERCIKTI
	Barış BAYKAL
	Uğur Bozlar
	Necdet Kocabıyık
	Mehmet Ali Güner
	Necati Salman
	Ayşe Özdemir
	Mislina Utlu
	Laçin Ramazanlı
**Additional Information:**	
**Question**	**Response**
**Financial Disclosure**<br><br>Enter a financial disclosure statement that describes the sources of funding for the work included in this submission. Review the submission guidelines for detailed	Yes

Powered by Editorial Manager® and ProduXion Manager® from Aries Systems Corporation

requirements. View published research articles from *PLOS ONE* for specific examples.<br><br>This statement is required for submission and **will appear in the published article** if the submission is accepted. Please make sure it is accurate.<br><br>**Funded studies**<br>Enter a statement with the following details:<br>• Initials of the authors who received each award<br>• Grant numbers awarded to each author<br>• The full name of each funder<br>• URL of each funder website<br>• Did the sponsors or funders play any role in the study design, data collection and analysis, decision to publish, or preparation of the manuscript?<br><br>Did you receive funding for this work?	
Please add funding details.<br>as follow-up to "**Financial Disclosure**<br><br>Enter a financial disclosure statement that describes the sources of funding for the work included in this submission. Review the submission guidelines for detailed requirements. View published research articles from *PLOS ONE* for specific examples.<br><br>This statement is required for submission and **will appear in the published article** if the submission is accepted. Please make sure it is accurate.	The budget of this research was covered by the Scientific Research Projects Coordination Unit of the University of Health Sciences within the scope of the project numbered 2023/022.

**Funded studies**<br>Enter a statement with the following details:<br>• Initials of the authors who received each award<br>• Grant numbers awarded to each author<br>• The full name of each funder<br>• URL of each funder website<br>• Did the sponsors or funders play any role in the study design, data collection and analysis, decision to publish, or preparation of the manuscript?<br><br>Did you receive funding for this work?"	
Please select the country of your main research funder (please select carefully as in some cases this is used in fee calculation).<br>as follow-up to "**Financial Disclosure**<br><br>Enter a financial disclosure statement that describes the sources of funding for the work included in this submission. Review the submission guidelines for detailed requirements. View published research articles from *PLOS ONE* for specific examples.<br><br>This statement is required for submission and **will appear in the published article** if the submission is accepted. Please make sure it is accurate.<br><br>**Funded studies**<br>Enter a statement with the following details:<br>• Initials of the authors who received each award<br>• Grant numbers awarded to each author<br>• The full name of each funder<br>• URL of each funder website<br>• Did the sponsors or funders play any role in the study design, data collection and analysis, decision to publish, or preparation of the manuscript?<br><br>Did you receive funding for this work?"	TURKEY - TR
**Competing Interests**	NO authors have competing interests

Use the instructions below to enter a competing interest statement for this submission. On behalf of all authors, disclose any competing interests that could be perceived to bias this work—acknowledging all financial support and any other relevant financial or non-financial competing interests.<br><br>This statement is required for submission and **will appear in the published article** if the submission is accepted. Please make sure it is accurate and that any funding sources listed in your Funding Information later in the submission form are also declared in your Financial Disclosure statement.<br><br>View published research articles from *PLOS ONE* for specific examples.<br><br>**NO authors have competing interests**<br><br>Enter: *The authors have declared that no competing interests exist.*<br><br>**Authors with competing interests**<br><br>Enter competing interest details beginning with this statement:<br><br>*I have read the journal's policy and the authors of this manuscript have the following competing interests: [insert competing interests here]*<br><br>* typeset	
**Ethics Statement**<br><br>Enter an ethics statement for this submission. This statement is required if the study involved:<br><br>• Human participants<br>• Human specimens or tissue<br>• Vertebrate animals or cephalopods<br>• Vertebrate embryos or tissues	This study was found ethically appropriate according to the Decision number 2023/83 dated 14.03.2023 of the Gülhane Scientific Research Ethics Committee of the University of Health Sciences. The need for informed consent was waived by the Gülhane Scientific Research Ethics Committee because the radiological data were analyzed retrospectively, and no personally identifiable information was accessed by the authors.

Powered by Editorial Manager® and ProduXion Manager® from Aries Systems Corporation

• Field research<br><br>Write "N/A" if the submission does not require an ethics statement.<br><br>General guidance is provided below. Consult the submission guidelines for detailed instructions. **Make sure that all information entered here is included in the Methods section of the manuscript.**	

Powered by Editorial Manager® and ProduXion Manager® from Aries Systems Corporation

**Format for specific study types**<br><br>**Human Subject Research (involving human participants and/or tissue)**<br>• Give the name of the institutional review board or ethics committee that approved the study<br>• Include the approval number and/or a statement indicating approval of this research<br>• Indicate the form of consent obtained (written/oral) or the reason that consent was not obtained (e.g. the data were analyzed anonymously)<br><br>**Animal Research (involving vertebrate animals, embryos or tissues)**<br>• Provide the name of the Institutional Animal Care and Use Committee (IACUC) or other relevant ethics board that reviewed the study protocol, and indicate whether they approved this research or granted a formal waiver of ethical approval<br>• Include an approval number if one was obtained<br>• If the study involved *non-human primates*, add *additional details* about animal welfare and steps taken to ameliorate suffering<br>• If anesthesia, euthanasia, or any kind of animal sacrifice is part of the study, include briefly which substances and/or methods were applied<br><br>**Field Research**<br><br>Include the following details if this study involves the collection of plant, animal, or other materials from a natural setting:<br>• Field permit number<br>• Name of the institution or relevant body that granted permission	
**Data Availability**<br><br>Authors are required to make all data underlying the findings described fully available, without restriction, and from the time of publication. PLOS allows rare exceptions to address legal and ethical concerns. See the PLOS Data Policy and FAQ for detailed information.	No - some restrictions will apply

Powered by Editorial Manager® and ProduXion Manager® from Aries Systems Corporation

A Data Availability Statement describing where the data can be found is required at submission. Your answers to this question constitute the Data Availability Statement and **will be published in the article**, if accepted.<br><br>**Important:** Stating 'data available on request from the author' is not sufficient. If your data are only available upon request, select 'No' for the first question and explain your exceptional situation in the text box.<br><br>Do the authors confirm that all data underlying the findings described in their manuscript are fully available without restriction?	
**Describe where the data may be found in full sentences. If you are copying our sample text, replace any instances of XXX with the appropriate details.**<br><br>• If the data are **held or will be held in a public repository**, include URLs, accession numbers or DOIs. If this information will only be available after acceptance, indicate this by ticking the box below. For example: *All XXX files are available from the XXX database (accession number(s) XXX, XXX.*).<br>• If the data are all contained **within the manuscript and/or Supporting Information files**, enter the following: *All relevant data are within the manuscript and its Supporting Information files.*<br>• If neither of these applies but you are able to provide **details of access elsewhere**, with or without limitations, please do so. For example:<br><br>*Data cannot be shared publicly because of [XXX]. Data are available from the XXX Institutional Data Access / Ethics Committee (contact via XXX) for researchers who meet the criteria for access to confidential data.*<br><br>*The data underlying the results presented in the study are available from (include the name of the third party*	The data underlying this study cannot be shared publicly due to confidentiality and ethical restrictions. This study was found ethically appropriate according to the Decision number 2023/83 dated 14.03.2023 of the Gülhane Scientific Research Ethics Committee of the University of Health Sciences. The data contain potentially identifiable information from cadaveric and radiological (patient X-ray) sources. Therefore, the data are not publicly available but can be accessed from the corresponding author upon reasonable request for researchers who meet the criteria for access to confidential data.

Powered by Editorial Manager® and ProduXion Manager® from Aries Systems Corporation

and contact information or URL).<br>• This text is appropriate if the data are owned by a third party and authors do not have permission to share the data.<br><br>* typeset	
Additional data availability information:	

Powered by Editorial Manager® and ProduXion Manager® from Aries Systems Corporation

Manuscript Click here to access/download;Manuscript;Paper.docx

A RESEARCH ARTICLE

PREVALENCE AND MORPHOMETRY OF THE FABELLA IN CADAVERIC AND RADIOLOGICAL STUDIES: IS THERE A LINK TO KNEE OSTEOARTHRITIS?

Ebru Yolaçan[1,*], Nurcan Ercıktı[2], Barış Baykal[3], Uğur Bozlar[4], Necdet Kocabıyık[2], Mehmet Ali Güner[2], Necati Salman[2], Ayşe Özdemir[3], Mislina Utlu[2], Laçin Ramazanlı[5]

[1] Samsun University Faculty of Medicine, Department of Anatomy, Samsun, Türkiye, https://orcid.org/0000-0001-8157-904X

* Corresponding author

[2] University of Health Sciences Gülhane Faculty of Medicine, Department of Anatomy, Ankara, Türkiye

Nurcan Ercıktı: https://orcid.org/0000-0002-7836-0095

Necdet Kocabıyık: https://orcid.org/0000-0003-2164-9231

Mehmet Ali Güner: https://orcid.org/0000-0002-9782-8044

Necati Salman: https://orcid.org/0000-0003-3927-8010

Mislina Utlu: https://orcid.org/0009-0002-0661-6837

[3] University of Health Sciences Gülhane Faculty of Medicine, Department of Histology and Embryology, Ankara, Türkiye

Barış Baykal: https://orcid.org/0000-0001-5668-2686

Ayşe Özdemir: https://orcid.org/0000-0002-4362-3959

[4] University of Health Sciences, Gülhane Faculty of Medicine, Department of Radiology, Ankara, Türkiye

Uğur Bozlar: https://orcid.org/0000-0002-2233-2079

[5] Azerbaijan Clinical Medical Center, Department of Radiology, Baku, Azerbaijan
Laçin Ramazanlı: https://orcid.org/0000-0002-4810-2925

ABSTRACT

Objective – To investigate the morphometric features of the fabella in cadaveric specimens and evaluate its prevalence and potential association with knee osteoarthritis through radiological analysis.

Methods – In the cadaveric study, 65 knees from 32 cadavers and 3 leg specimens were dissected. Fabellae were measured and analyzed histologically. In the radiological study, bilateral knee X-rays of 1712 patients with osteoarthritis and 2304 control subjects were retrospectively reviewed. The presence and laterality of the fabella were recorded.

Results – Fabella was detected in 18.5% of dissected knees. No significant sex-based differences were found in morphometric measurements (p>0.05). In the radiological study, the overall prevalence was 36.5% (37.6% in patients, 35.7% in controls), with no statistically significant association between fabella presence and osteoarthritis (p>0.05). Lateralization patterns did not differ between groups.

Conclusion – This study provides the first morphometric data on fabellae in Turkish cadavers and suggests no significant relationship between fabella presence and knee osteoarthritis. While fabella is a common anatomical variant, its clinical relevance in osteoarthritis remains inconclusive, warranting further investigation.

Keywords: fabella, sesamoid bone, Turkish population, osteoarthritis.

1. INTRODUCTION

The fabella is a sesamoid ossicle typically located within the tendon of the lateral head of the gastrocnemius muscle. However, it has been demonstrated that it can also be found in the tendon of the medial head, although rarely. The fabella contributes to minimizing frictional injury to the tendon, enhances the mechanical efficiency of the gastrocnemius muscle, and plays a role in stabilizing the posterolateral aspect of the knee joint through its interaction with the fabellofibular ligament [1-4]. If fabella is absent do you think the above functions are compromised?

Fabella occurs in 10-30% of the general population. It is frequently observed bilaterally.

Merge this sentence with the following one

In the literature, it is generally reported to be more common in Asia [5-7]. Current studies in the literature indicate that fabella is common in Asian countries [3,6]. This is thought to be due to cultural differences. Kneeling and squatting are common in Asian societies. Due to this traditional way of sitting, the fabella undergoes permanent compression on the posterior aspect of the lateral condyle of the femur, which accelerates ossification of the fabella [8,9].

Fabella is usually detected incidentally on radiographs. Yet, depending on its location or osteophytization, fabella may cause atypical pain in the posterolateral region of the knee. Clinically, it leads to pathologies such as fracture, fabella syndrome, nervus fibularis communis paralysis, chondromalacia, and osteoarthritis [7,10,11].

In recent years, it has been revealed that the relationship between fabella and clinical conditions is significant [6,12]. For this reason, studies on fabella and clinical conditions have started to increase. Undoubtedly, the most striking of these clinical conditions is the relationship between knee osteoarthritis and the presence of fabella, which is still unclear. In Prittchett's study, when he compared the radiographs of individuals with osteoarthritis in the knee and normal individuals, he found that fabella was more likely in the osteoarthritic knee [13].

This study includes a two modality research pattern. The cadaveric part aimed to analyze the morphometric properties of fabellae obtained after dissection, while the radiological part

gender

aimed to examine the prevalence of fabellae, lateralization pattern, genders distribution and

potential correlation between the presence of fabellae and osteoarthritis in the Turkish population.

2. MATERIALS AND METHODS

This study was found ethically appropriate according to the Decision number 2023/83 dated 14.03.2023 of the Gülhane Scientific Research Ethics Committee of the University of Health Sciences. The need for informed consent was waived by the Gülhane Scientific Research Ethics Committee because the radiological data were analyzed retrospectively, and no personally identifiable information was accessed by the authors.

Cadaveric dissections were carried out in the Anatomy Laboratory of Gülhane Faculty of Medicine between 20/03/2023 and 27/03/2023. The specimens were delivered to the Department of Histology and Embryology on 29/03/2023, and the histological analysis results were reported to the researchers on 29/05/2023.

For the radiological study, retrospective knee radiographs covering the period between 01/01/2020 and 31/12/2021 were included. These data were accessed and analyzed between 20/03/2023 and 20/05/2023.

2.1. CADAVERIC STUDY

Six Three

In our study, 32 formaldehyde-fixed cadavers (6 females, 26 males) and 3 male

How do you ascertain the sex by leg to

specimens (2 left legs, 1 right leg) aged between 60 and 85 years were dissected in the anatomy dissection laboratory of Gülhane Medical Faculty, University of Health Sciences. The right legs

two

of both female cadavers were amputated. The lateral and medial heads of the gastrocnemius

Identifie

muscle were palpated by dissection in the posterior region of the knee and fibrocartilaginous structures were investigated. In suspicious cases, the capsule was incised and condyles of the femur were exposed. The excised fabellae were stored in 10% formaldehyde solution and separated from the surrounding tissues under a dissecting microscope (Carl ZEISS, Germany; 4.0-6.0× magnification). Superior Inferior (SI)-Anterior Posterior (AP) dimensions were

measured with digital calipers by two independent investigators (Figure 1). The specimens were then transferred to the Gülhane Medical Faculty Department of Histology and Embryology for histopathologic analysis.

Figure 1: Fabella measurements were performed by two-dimensionally. (A) SI measurement with caliper. (B) AP measurement with caliper. (C) Collective view of the removed Fabella.

The fabellae were decalcified in 20% EDTA solution for eight 8 days. After decalcification, 7-µm-thick sections were prepared using a cryotome and dried on slides overnight. Histological evaluation was performed using Hematoxylin-Eosin and Masson's Trichrome staining protocols.

2.2. RADIOLOGIC STUDY

The radiologic part of the study was conducted using the image database of the Department of Radiology of Gülhane Training and Research Hospital, Ankara, Türkiye, between the dates of January 2020 and December 2021. Radiological data from this period were retrospectively analyzed, and no personally identifiable information was accessed by the authors. Patients group composed of patients over 40 years, had bilateral (anterior-posterior/lateral) knee roentgenograms, and were diagnosed with knee osteoarthritis. The control group composed of radiology department admissions with the same age group and had bilateral knee roentgenograms throughout the same time period. Patients with suboptimal image quality, non-standard posture, unilateral images, and the presence of duplicate records were excluded. During the initial assessment, 2246 patients and 3136 healthy individuals were identified, Consequently, 1712 patients and 2304 controls were enrolled in the study. In all

cases, the presence of the fabella was independently confirmed by two radiologists and two anatomists (Figure 2).

Figure 2: A bilateral lateral knee roentgenogram showing the fabellae, each surrounded by an elliptical circle, in both the right and left legs.

2.3. STATISTICAL ANALYSIS

In the cadaver study, descriptive statistics were evaluated concerning gender, the number of fabellae, measurements, and categorical data presented as frequency (n) and percentage (%). In the radiology study, calculated the sample size based on a statistical power of 80% and a Type 1 error of 5%. The normal distribution of continuous variables was analyzed by Kolmogorov-Smirnov (n>50) and Skewness-Kurtosis tests. Independent Samples T-test was applied for continuous variables in comparisons between groups, and Chi-square (χ^2) test was used to evaluate the relationships between categorical variables. The statistical significance level was taken as $p<0.05$ in the calculations and SPSS (IBM SPSS for Windows, version 26.0 software) statistical package program was used for the analyses.

3. RESULTS

3.1. CADAVERIC STUDY

Since amputee legs and specimen tissues were in our sample, the prevalence calculation was based on the total number of knees. Fourteen fabella, all of which were excised from the tendon of the lateral head of the gastrocnemius muscle were sent for histopathologic examination. It was determined that 2 two of these structures were muscular tissue and 12 were bony fabellae (Figure 3). Therefore, the measurement results of 12 fabella were included to

statistical analysis. During dissections, traces of three fabellae were found on the lateral condyle of the femur. These traces were named sesamoid traces made by the fabella (Figure 4).

Figure 3: (A) H&E-stained fabella section showing the tendon (T) attachment to compact bone (CB) with a fibrocartilage (FC) transition zone. (B) Trabecular bone within the fabella, stained with Masson's trichrome, revealing a central vascular canal (VC) that facilitates nutrient supply to bone tissues.

Figure 4: Fabella sesamoid traces with fabellas. (A) Medial head of the Gastrocnemius muscle (GC-CM) and lateral head of the Gastrocnemius muscle (GC-CL) in the right leg of a mature male cadaver, with the common fibular nerve (CFN) visible. (B) The left leg of a mature female cadaver. (C) GC-CM and GC-CL in the left leg of a mature female cadaver, with the CFN and Soleus muscle (S) visible.

In 65 dissected knees, 12 fabella were found. The total prevalence of fabella was 18.4%. Three fabella were found in 27 male right legs and one fabella was found in 4 female right legs. Six fabella were found in 28 male left legs and two fabella were found in 6 female left legs. The prevalence of fabella between males and females was not statistically significant ($p>0.05$). When the SI and AP measurements of the right and left fabella were analyzed, although the fabella measurements of female were slightly higher than those of men, there was no significant statistical difference between the SI and AP measurements of male and female ($p>0.05$).

3.2. RADIOLOGIC STUDY

The study was conducted with data collected from 1712 patients (42.6%) and 2304 healthy individuals (57.4%) who were admitted to the Radiology Clinic of Gülhane Training and Research Hospital, University of Health Sciences. In the evaluation of the participants, the gender distribution of the healthy and patients; for the patient group; 79.2% (n: 1356) of the study were female and 20.8% (n: 356) were male, while in the control group; 75.2% (n: 1733) of the study were female and 24.8% (n: 571) were male. The mean±standard deviation of the age of the participants was for the patient group; 63.48 ±9.95 (minimum age: 40 years, maximum: 96 years, Median: 63), for the control group; 58.84 ±11.06 (minimum age: 40 years, oldest age: 98, Median value: 58). The total prevalence of fabella was 36.5%, the prevalence of female fabella was 35.6%, and the prevalence of male fabella was 39.5%. No statistically significant sex-based difference was observed in the prevalence of the fabella (p>0.05) (Table 1).

Table 1: Prevalences of Fabella for Female and Male.

		Gender						
		Female		Male			Total	Chi-square
		n	%	n	%	n	%	p
Prevalence of Fabella	The presence of a fabella in any leg	1099	35,6	366	39,5	1465	36,5	0.463
	Absent	1990	64,4	561	60,5	2551	63,5	
	Total	3089	100,0	927	100,0	4016	100,0	

The presence of fabella on the right and left legs of the patients and healthy individuals is given in Table 2 and the rate of presence of fabella is 37.6% in patients and 35.7% in healthy
patient
individuals. The prevalence of fabella in patients individuals did not differ significantly from those who were healthy (p>0.05). Statistical analysis revealed no significant difference between the groups in terms of laterality (side) (p > 0.05). In other words, we can say that the one-sided or double-sided fabella variable does not change according to patients or healthy people.

Table 2: Presence of fabella on the right and/or left legs of patient and healthy individuals.

		Patient		Healthy		Total		Chi-squared test	
		n	%	n	%	n	%	χ^2	p
Fabella in the Right Leg	Absent	1290	75,4	1723	74,8	3013	75,0	0,169	0,681
	Present	422	24,6	581	25,2	1003	25,0		
Fabella in the Left Leg	Absent	1181	69,0	1630	70,7	2811	70,0	1,453	0,228
	Present	531	31,0	674	29,3	1205	30,0		
Fabella	Absent	1069	62.4	1482	64.3	2551	63.5	1.50	0.221
	Present	643	37.6	822	35.7	1465	36.4		
Side	Unilateral	333	19.4	389	16.9	722	18	2.88	0.090
	Bilateral	310	18.1	433	18.8	743	18.5		

** Significance levels according to chi-square test results*

***Significance levels according to independent T-test results*

4. DISCUSSION

4.1. CADAVERIC STUDY

Located within the tendon of the lateral head of the gastrocnemius muscle, the fabella is a sesamoid bone that generally remains asymptomatic and is often discovered incidentally during imaging. It is usually detected by radiologic examinations or incidentally in cadaver dissection studies. The methods used in prevalence studies significantly affect the results. Especially X-ray based imaging techniques, roentgenography and computed tomography, may overlook cartilaginous fabellae. This leads to significant differences in prevalence rates in the literature [14]. In the dissection study conducted by Zeng et al., 11 of 19 the cartilaginous fabella identified by palpation could not be visualized on radiographs [2]. In another study by Chew et al., radiographs and magnetic resonance imaging scans of 80 patients were followed to determine the presence of fabellae, but cartilage fabellae detected on magnetic resonance imaging could not be detected on radiography [8].

In cadaveric studies, the prevalence of fabella was generally found to be higher, with the highest rates reported especially in Asian populations (Table 3).

Table 3: Cadaver study-based prevalence values reported in the literature.

Prevalence

Researchers	Year	Population	Method	Age	Fabella/Knee	Prevalans
Chihlas et al. [15]	1993	United States	Cadaver	-	18/78	%27
Minowa et al. [16]	2004	Japan	Cadaver	54-98	182/212	%85.8
Kawashima et al. [1]	2007	Japan	Cadaver	66-100	99/150	%66.0
Raheem et al. [17]	2007	Ireland	Cadaver	84±8.1	2/22	%9.1

Silva et al. [18]	2010	Brazil	Cadaver	38-78	2/62	%3.1
Phukubye and Oyedele [19]	2011	Africa	Cadaver	40-95	12/102	%11.7
Tabira et al. [3]	2012	Japan	Cadaver	74.5±12.3	70/102	%68.6
Piyawinijwong et al. [20]	2012	Thailand	Cadaver	30-97	144/372	%38.7
Mohite et al. [21]	2016	India	Cadaver	-	5/40	%12.5
Present Study	2023	Türkiye	Cadaver	60-85	12/65	%18.4

In this present study, the total prevalence was calculated as 18.4% (12/65) and no significant difference was found between genders ($p>0.05$). As far as is known, no other prevalence of cadaver studies has been reported from Türkiye. In the research carried out by Phukubye et al., the total prevalence of fabella was found to be 11.7% and no meaningful variation was detected between male and female participants ($p>0.05$). From this point of view, similar findings have emerged with our study [19]. Tabira et al. reported a much higher prevalence (68.6%) but did not reveal a considerable variation between genders [3].

Although fabellae have been reported in the medial gastrocnemius head tendon, all specimens dissected in this study were located in the lateral head tendon. In the paper of Phukubye et al., all fabellae were located in the tendon at the lateral head of the gastrocnemius, which is consistent with our study in this respect [19]. Zeng et al. and Kawashima et al. reported the presence of fabellae in the medial head of the gastrocnemius muscle [1,2]. In this regard, it contrasts with our findings.

In the lateral condyle region of the femur, traces of sesamoid bones were detected. Kawashima et al. reported that the bony fabella left trace on the femoral condyle [1]. Tabira et al. found a round depression in this region [3]. Zeng et al. reported that bony and cartilaginous fabellae formed traces on the femoral condyle [2].

In our work, due to comparing fabella measurements according to gender, the right fabella length (SI) was found to be 10.46±1.19 mm in males and 10.90 mm in females. Right fabella thickness (AP) was 4.23±1.42 mm in males and 3.95 mm in females. Left fabella SI was 10.83±0.68 mm in males and 12.45±1.39 mm in females; left fabella AP was 4.02±0.69 mm in males and 4.49±0.77 mm in females. While fabella measurements were generally slightly higher in females, statistical analysis revealed no notable gender-based differences ($p > 0.05$).

In general females bone are said to be smaller than males. How would you justify the reason for more values in female

Hauser et al. reported SI values similar to our study (3.63-11.71 mm), although they did not provide data on gender distributions [22], whereas Chew et al. reported an average SI of 7.50 ± 1.91 mm and AP of 4.89 ± 1.97 mm for male and SI of 5.86 ± 0.99 mm and AP of 4.65 ± 1.94 mm for female. In addition, no relationship was found between gender and fabella dimensions. These data partially coincide with our results [8].

4.2. RADIOLOGY STUDY

The prevalence of fabella exhibits variation across populations, influenced by factors such as ethnicity and geographical distribution. Moreover, this prevalence may also differ within distinct regions of the same country (Table 4).

Table 4: Radiology study-based prevalence values reported in the literature.

Prevalence

Researchers	Year	Population	Method	Age	Fabella/Knee	Prevalans
Yu et al. [23]	1996	United States	Radiography (MRI)	12-72	19/100	19.0%

Sarin et al. [24]	1999	United States	Radiography (Direct Radiography)	19-84	55/224	31.3%
Zeng et al. [2]	2012	China	Cadaver+ Radiography	64-83	53/61	86.9%
Chew et al. [8]	2014	Singapore	Radiology (Knee arthroscopy, MRI)	14-55	25/80	31.3%
Ehara [25]	2014	Japan	Radiography (MRI)	4-89	200/653	30.6%
Hauser et al. [22]	2015	Switzerland	Radiology (CT)	20-104	105/400	26.3%
Egerci et al. [4]	2016	Türkiye	Radiography (Direct Radiography)	18-90	228/1000	22.8%
Ghimire et al. [26]	2017	Nepal	Radiography (Direct Radiography)	-	19/155	12.3%
Hedderwick et al. [27]	2017	New Zealand	Radiology (MRI)	Mean age 23 ± 3.2 years	8/28	28.5%
Pop et al. [28]	2018	Romania	Radiology (MRI)	21-43	73/862	8.4%
Berthaume et al. [29]	2019	Korea	Radiology (CT)	21-60	94/212	44.34%

Hou et al. [10]	2019	China	Radiography (Direct Radiography)	21months-86 years	660/1359	48.6%
Hur et al. [12]	2020	Korea	Radiography (Direct Radiography)	18^{+}	2172/4252	51.1%
Adedigba et al. [9]	2021	Africa	Radiography (Direct Radiography)	3-100	77/754	10.2%
Sari et al. [6]	2021	Türkiye	Radiography (Direct Radiography)	Female Mean±SD 61.33±12.38 Male Mean±SD 59.28±15.25	380/2000	19%
Akkoç et al. [30]	2022	Türkiye	Radiology (MRI)	18^{+}	605/2035	29.7%
Zhong et al. [31]	2022	Korea	Radiology (MRI)	8-91	402/1011	39.8%
Present Study	2023	Türkiye	Radiography (Direct Radiography)	40-98	1465/8032	18.2%
Packirisamy et al. [32]	2024	Saudi Arabia	Radiology (MRI)	20-90	170/820	20.73%

Shete et al. [33]	2024	India	Radiography (Direct Radiography)	18^{+}	87/500	17.40%

In the study by Sarı et al. the prevalence of fabella was 24.3% in general, 25.8% in female and 20.6% in male [6]. Egerci et al. reported these rates as 22.8%, 24% and 21.6%, respectively [4]. Akkoç et al. found the prevalence of fabella to be 39.6% in female and 38.4% in male, with a general prevalence of 38.8% [30]. The findings of Özbay et al. show an overall prevalence of 20.63%, 21.21% in male and 20.27% in female [34]. In our study, the prevalence of fabella was 36.5%, 35.6% for female and 39.5% for male. While the results of Akkoç et al. support our findings [30], they differ from the results of other studies, suggesting that the prevalence of fabella may show regional differences within the same country.

Özbay et al., Ünlütürk et al., Egerci et al. Sarı et al., Hur et al. did not detect a statistically significant difference between gender and prevalence of fabella [4,6,12,34,35]. According to a recent meta-analysis by Berthaume et al., fabella prevalence rates were 1.32-2.60% more prevalent in male than female in the last 143 years, and 2.47-2.60% more prevalent in 2018 [14]. This has been explained by the ossification of sesamoid bones as a result of high mechanical stimuli and the lateral head of the gastrocnemius being under more tension in male. However, the current study, along with in other papers conducted in the Turkish population, there was no statistically significant difference in the prevalence of fabella between male and female ($p>0.05$).

The findings of our study indicate that there was no statistically significant difference in the prevalence of fabella between individuals with osteoarthritis (37.6%) and in healthy individuals (35.7%) ($p>0.05$). This finding is inconsistent with the results of Pritcett's study and the meta-analysis of Asghar et al. suggesting a positive association between osteoarthritis and

the presence of fabella [13,36]. This could possibly be due to our sample characteristics (predominance of early-stage osteoarthritis) or multifactorial mechanisms in the formation of the fabella.

This research, the prevalence of unilateral and bilateral fabellae in the patient group was 19.4% and 18.1%, respectively, while these rates were 16.9% and 18.8%, respectively, in the healthy group. The total unilateral prevalence was 18% and bilateral prevalence was 18.5%. The present study, there was no statistically significant difference between the patient and healthy groups. That is to say, we found that the variable of unilateral or bilateral fabella did not differ between patients and healthy subjects. Yet, in the laterality analysis, it was determined that right-sided fabellas were mostly bilateral, while left-sided ones had a lower percentage of bilateral fabella than the right, although the percentage of fabella appeared higher. According to Sari et al., the prevalence of unilateral or bilateral fabella on the left side was significantly greater among patients diagnosed with osteoarthritis than in those without osteoarthritic changes [6]. In a meta-analysis paper, Asghar et al. found that the prevalence of bilateral fabella was higher than unilateral fabella, and the prevalence of fabella was higher on the right side in unilateral and bilateral prevalence [36]. The differences in prevalence are probably due to heterogeneity in the study populations. This discrepancy in laterality distribution may be related to dominant joint use or asymmetric load distribution.

The limitation of this research at the cadaver stage is that the fabella palpated manually before dissection were not visualized radiologically. If radiological examination could be performed, the presence of fabella would have been confirmed by imaging methods and the evidentiary value of the findings would have increased. Nevertheless, due to the lack of opportunity to perform radiographic imaging on cadavers, this verification was not feasible. Our limitation at the radiology part is that since only radiographic imaging was used, fabella with a cartilaginous structure could not be detected. Therefore, our findings only reflect the

prevalence of ossified fabella. Although magnetic resonance imaging (MRI) will allow for more detailed evaluation of the fabella and surrounding tissues, the cost-effectiveness of this method is debatable.

5. CONCLUSION

The fabella, a sesamoid bone of the knee joint, continues to attract anatomical interest due to its variable presence, morphology, and clinical implications. To our knowledge, this is the first study in Türkiye to document the prevalence and morphological characteristics of cadaveric fabellae using histological staining. Notably, our radiological findings revealed no significant association between fabella presence and knee osteoarthritis—a result that contrasts with several previous reports. This divergence highlights the complexity of the fabella's role in joint pathology and underscores the necessity of broader, population-specific investigations to elucidate its clinical relevance more accurately.

ACKNOWLEDGMENTS

This study was produced from the medical specialization thesis titled ‘Morphometry of Fabella in Human Cadaver and Radiological Evaluation of Fabella in Osteoarthritis Cases’ which was conducted under the supervision of Assoc. Prof. Nurcan Ercıktı, with co-researchers from different medical disciplines who are experienced in their fields, and defended by Dr. Ebru Yolaçan on 15.06.2023.

FUNDING INFORMATION

The budget of this research was covered by the Scientific Research Projects Coordination Unit of the University of Health Sciences within the scope of the project numbered 2023/022.

AUTHOR CONTRIBUTIONS:

Conception and design of the study: Ebru Yolaçan; Nurcan Ercıktı; Necdet Kocabıyık; Mehmet Ali Güner; Definition of intellectual content: Ebru Yolaçan; Literature search: Ebru Yolaçan; Clinical studies: Uğur Bozlar; Experimental studies (cadaveric and histological): Ebru Yolaçan; Barış Baykal; Ayşe Özdemir; Necdet Kocabıyık; Necati Salman; Radiological data analysis and classification: Ebru Yolaçan; Laçin Ramazanlı; Mislina Utlu; Uğur Bozlar; Statistical analysis: Mehmet Ali Güner; Manuscript preparation: Ebru Yolaçan; Nurcan Ercıktı; Manuscript editing and review: Ebru Yolaçan; Nurcan Ercıktı; Barış Baykal; Uğur Bozlar; Laçin Ramazanlı; Mislina Utlu; Necati Salman; Mehmet Ali Güner; Ayşe Özdemir; Necdet Kocabıyık.

CONFLICT OF INTEREST

The authors declare that they have no conflict of interest.

REFERENCES

1. Kawashima T, Takeishi H, Yoshitomi S, Ito M, Sasaki H. Anatomical study of the fabella, fabellar complex and its clinical implications. Surg Radiol Anat. 2007;29(7):611–6.
2. Zeng SX, Dong XL, Dang RS, Wu GS, Wang JF, Wang D, et al. Anatomic study of fabella and its surrounding structures in a Chinese population. Surg Radiol Anat. 2012;34(1):65–71.
3. Tabira Y, Saga T, Takahashi N, Watanabe K, Nakamura M, Yamaki KI. Influence of a fabella in the gastrocnemius muscle on the common fibular nerve in Japanese subjects. Clin Anat. 2013;26(7):893–902.

4. Egerci OF, Kose O, Turan A, Kilicaslan OF, Sekerci R, Keles-Celik N. Prevalence and distribution of the fabella: a radiographic study in Turkish subjects. Folia Morphol (Warsz). 2017;76(3):478–83.
5. Dalip D, Iwanaga J, Oskouian RJ, Tubbs RS. A comprehensive review of the fabella bone. Cureus. 2018;10(6):e2736.
6. Sari A, Dincel YM, Cetin MU, Gunaydin B, Guney M. The prevalence of fabella in Turkish population and the association between the presence of fabella and osteoarthritis. SN Compr Clin Med. 2021;3(3):805–11.
7. Woliński F, Byliński Ł, Kostelecka K, Teresiński G, Buszewicz G, Baj J. Common fibular nerve palsy due to the fabella: A review. Clin Anat. 2024;37(1):73–80.
8. Chew CP, Lee KH, Koh JSB, Howe TS. Incidence and radiological characteristics of fabellae in an Asian population. Singapore Med J. 2014;55(4):198–201.
9. Adedigba JA, Idowu BM, Hermans SP, Okwori OF, Onigbinde SO, Oluwadiya KS, et al. Fabella and patella variants: radiographic prevalence, distribution and clinical relevance in a population of black African descent. Anat Cell Biol. 2021;54(2):184–92.
10. Hou W, Xu L, Wang J, Wang B, Liu L, Xu K. Fabellar prevalence, degeneration and association with knee osteoarthritis in the Chinese population. Sci Rep. 2019;9(1):13046.
11. Diaz CC, Agarwalla A, Forsythe B. Fabella syndrome and common peroneal neuropathy following total knee arthroplasty. Case Rep Orthop. 2021;2021:1–4.
12. Hur JW, Lee S, Jun JB. The prevalence of fabella and its association with the osteoarthritic severity of the knee in Korea. Clin Rheumatol. 2020;39(12):3625–9.
13. Pritchett JW. The incidence of fabellae in osteoarthrosis of the knee. J Bone Joint Surg Am. 1984;66(9):1379–80.

14. Berthaume MA, Bull AMJ. Human biological variation in sesamoid bone prevalence: the curious case of the fabella. J Anat. 2020;236(2):228–42.
15. Chihlas CN, Ladocsi LT, Sholley MM, Loughran TP, Krieg RJ Jr. Position of the fabella relative to the path of the common peroneal nerve across the lateral head of the gastrocnemius muscle. Clin Anat. 1993;6(3):163–6.
16. Minowa T, Murakami G, Kura H, Suzuki D, Han SH, Yamashita T. Does the fabella contribute to the reinforcement of the posterolateral corner of the knee by inducing the development of associated ligaments? J Orthop Sci. 2004;9(1):59–65.
17. Raheem O, Philpott J, Ryan W, O'Brien M. Anatomical variations in the anatomy of the posterolateral corner of the knee. Knee Surg Sports Traumatol Arthrosc. 2007;15(8):895–900.
18. Silva JG, Chagas CAA, Torres DFM, Servidio L, Vilela AC, Chagas WA. Morphological analysis of the fabella in Brazilians. Int J Morphol. 2010;28(1):105–10.
19. Phukubye P, Oyedele O. The incidence and structure of the fabella in a South African cadaver sample. Clin Anat. 2011;24(1):84–90.
20. Piyawinijwong S, Sirisathira N, Sricharoenvej S. The fabella, fabellofibular and short lateral ligaments: an anatomical study in Thai cadavers. Siriraj Med J. 2012;64(Suppl 1):S15–8.
21. Mohite S, Mohite H, More R. Morphological study of the plantaris muscle. Int J Health Sci Res. 2016;6(1):125–30.
22. Hauser NH, Hoechel S, Toranelli M, Klaws J, Müller-Gerbl M. Functional and structural details about the fabella: what the important stabilizer looks like in the central European population. Biomed Res Int. 2015;2015:343728.

23. Yu JS, Salonen DC, Hodler J, Haghighi P, Trudell D, Resnick D. Posterolateral aspect of the knee: improved MR imaging with a coronal oblique technique. Radiology. 1996;198(1):199–204.
24. Sarin VK, Erickson GM, Giori NJ, Bergman AG, Carter DR. Coincident development of sesamoid bones and clues to their evolution. Anat Rec. 1999;257(5):174–80.
25. Ehara S. Potentially symptomatic fabella: MR imaging review. Jpn J Radiol. 2014;32(1):1–5.
26. Ghimire I, Maharjan S, Pokharel GB, Subedi K. Evaluation of occurrence of sesamoid bones in the lower extremity radiographs. J Chitwan Med Coll. 2017;7(2):11–4.
27. Hedderwick M, Stringer MD, McRedmond L, Meikle GR, Woodley SJ. The oblique popliteal ligament: an anatomic and MRI investigation. Surg Radiol Anat. 2017;39(10):1017–27.
28. Pop TS, Pop AM, Olah P, Trâmbiţaş C. Prevalence of the fabella and its association with pain in the posterolateral corner of the knee: a cross-sectional study in a Romanian population. Medicine (Baltimore). 2018;97(47):e13333.
29. Berthaume MA, Di Federico E, Bull AMJ. Fabella prevalence rate increases over 150 years, and rates of other sesamoid bones remain constant: a systematic review. J Anat. 2019;235(1):67–79.
30. Akkoc RF, Aksu F, Emre E, Sap O, Karatas A, Aydin S. The morphology of fabella and its prevalence in Turkish society. Eur Rev Med Pharmacol Sci. 2022;26(4):1164–9.
31. Zhong J, Zhang G, Si L, Hu Y, Xing Y, He Y, et al. The prevalence and parameters of fabella and its association with medial meniscal tear in China: a retrospective study of 1011 knees. BMC Musculoskelet Disord. 2022;23(1):188.

32. Packirisamy V, Aljarrah K, Tasslaq SE, Saikarthik J, Nayak SB. Prevalence and radiological features of fabella in a Saudi Arabian population. Muscles Ligaments Tendons J. 2024;14(2):263–9.
33. Shete S, Khanapure H, Moulick A. Radiological study of ossified fabella in Indian knees: a cross-sectional study. J Clin Diagn Res. 2024;18(6):1–4.
34. Özbay H, Mraja HM, Can A, Erdoğan F, Mraja HM. Prevalence and radiological characteristics of the fabella in Turkish population. Cureus. 2022;14(11):e12345.
35. Unluturk O, Duran S, Teke YH. Prevalence of the fabella and its general characteristics in Turkish population with magnetic resonance imaging. Surg Radiol Anat. 2021;43(12):2047–54.
36. Asghar A, Naaz S, Narayan RK, Kumar A. Does the prevalence of ossified fabella vary in knee osteoarthritis and age-related degeneration? A meta-analysis of about 11,000 knees. Cureus. 2021;13(1):e1234.

 Click here to access/download;Figure;Figure-1.jpeg

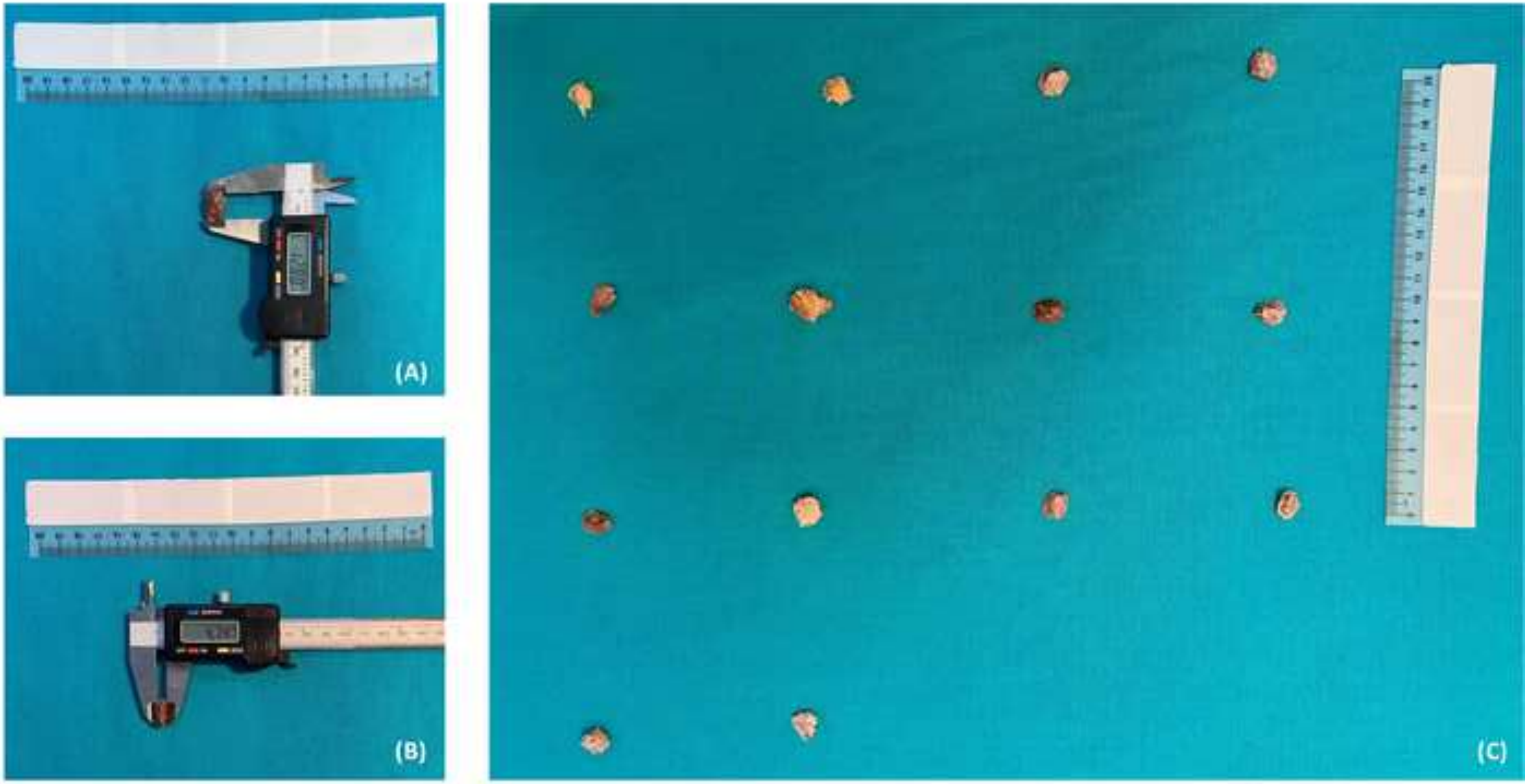

Click here to access/download;Figure;Figure-2.jpeg

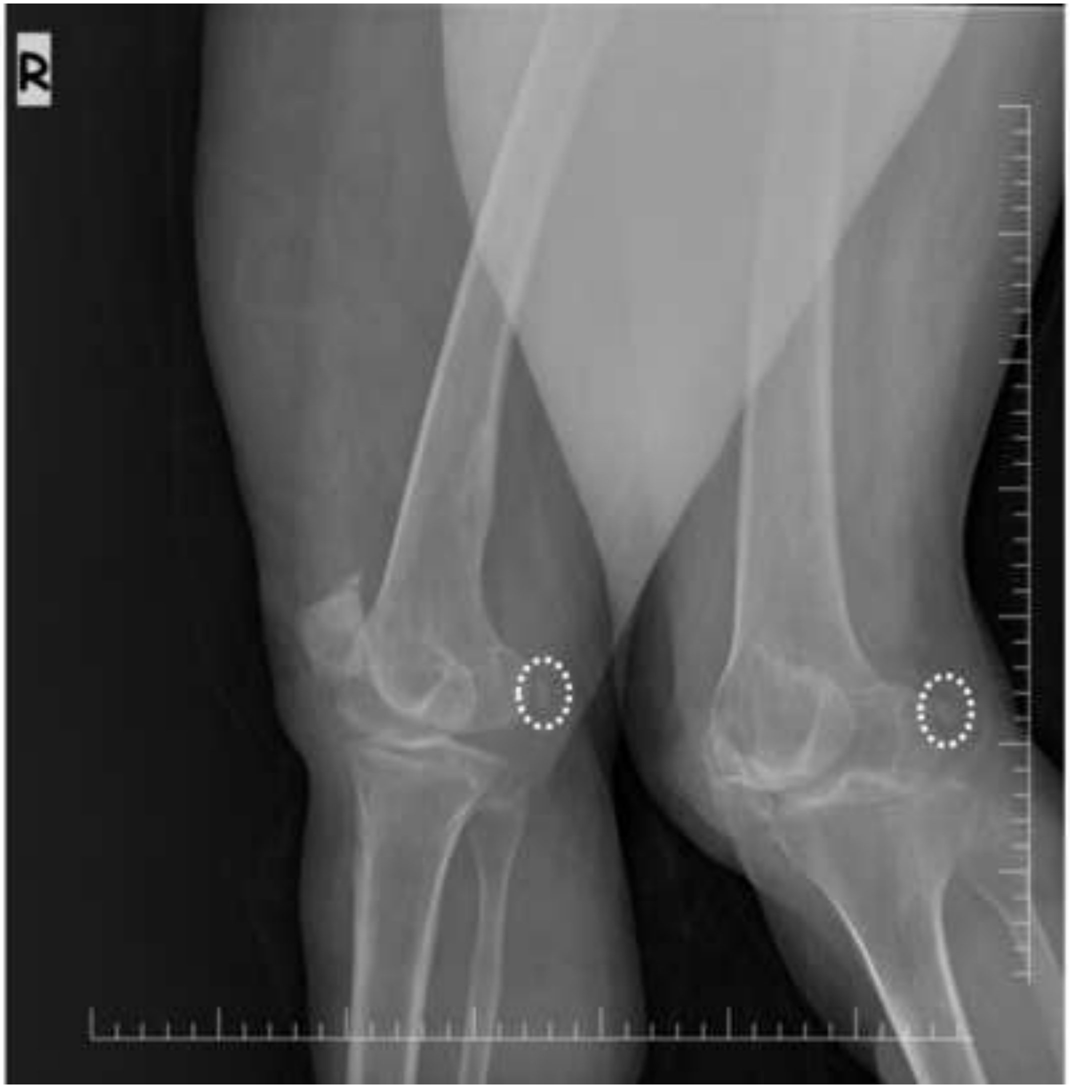

Click here to access/download;Figure;Figure-3.jpeg

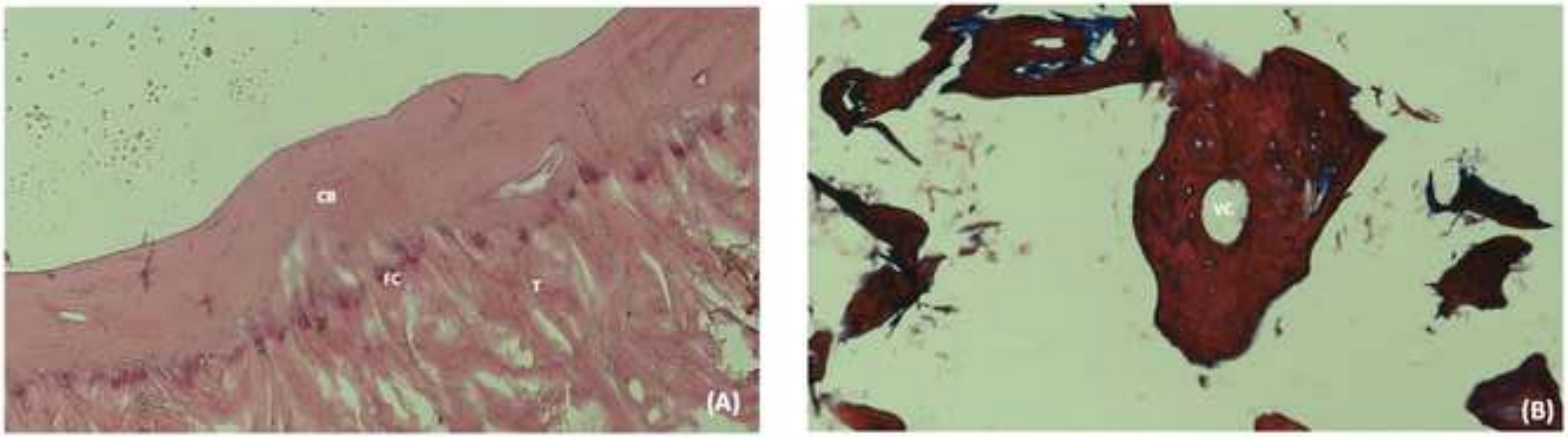

Click here to access/download;Figure;Figure-4.jpeg

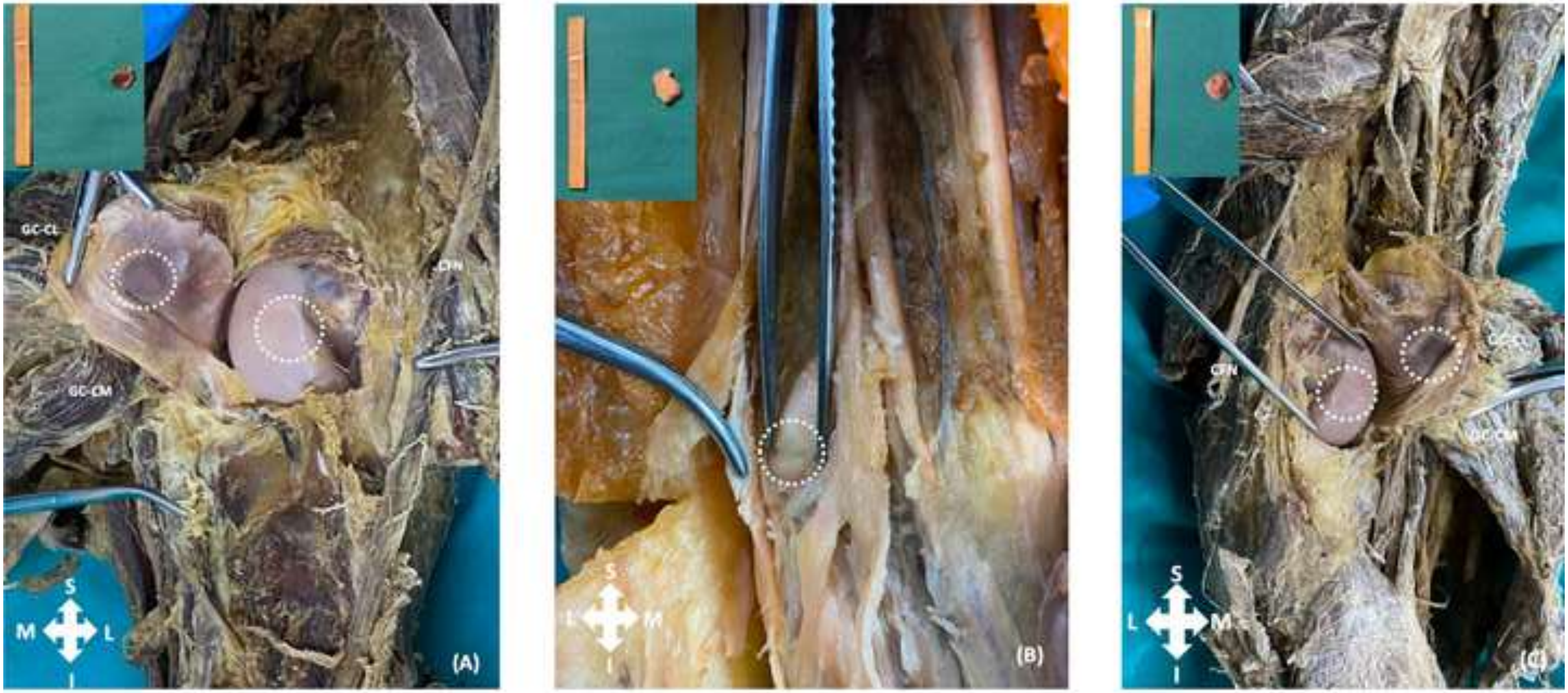

Click here to access/download
Supporting Information
First Page File.docx

Ethics Approval Translation

Click here to access/download

Supporting Information

Ethics_Approval_Translation.docx
