# Supplementary material for: Prevalence and morphometry of the fabella in cadaveric and radiological studies: Is there a link to knee osteoarthritis?
Source: PLoS One. 2026 Jan 5;21(1):e0340399. doi: 10.1371/journal.pone.0340399 (PMC12768361; doi:10.1371/journal.pone.0340399)
Supplement: Attachment — Submitted filename: Review report_PLOS.docx [file pone.0340399.s005.docx]

**Review report:**

**PREVALENCE AND MORPHOMETRY OF THE FABELLA IN CADAVERIC AND**

**RADIOLOGICAL STUDIES: IS THERE A LINK TO KNEE OSTEOARTHRITIS?**

The prevalence and morphometry of the fabella in a Turkish population, as well as any possible connections to osteoarthritis (OA), are discussed in this study that combines cadaveric, histological, and radiographic investigations.

To improve readability, flow, and clarity, however, some small changes are required, particularly in the Introduction, Results, and Discussion sections. Minor terminology and statistical expression errors should be fixed, and certain phrases can be made shorter.

### Introduction:

- Condense the repeated statements about fabella prevalence and Asian populations.
- Clarify cultural reasoning: why the higher prevalence in Asian populations is thought to be associated with habitual kneeling and squatting postures and which may increase mechanical stimulation and ossification of the fabella.”
- Last paragraph can be ended with more clearer research aim /objectives

### Results

- Provide confidence intervals (if available) for key prevalence figures.
- Add a brief linking statement before each table to improve flow, For example-Table 1 summarizes the distribution of fabella by sex.
- Use table only legends when it is shown in the table (such as asterisks in table-2 used in the legend, but it is not shown in the content values)
- In table -3, use of symbol % - should be in consistent with that of text. Generally it is used after number (eg. 14%.... ) Kindly check this

### Discussion

Some paragraphs repeat details from the Results. Avoid repetition to make it concise and consider to condense to focus on interpretation.

You may add a paragraph explicitly discussing **possible biomechanical explanations** for the lack of association between fabella and OA.
